# An island of wildlife in a human-dominated landscape: The last fragment of primary forest on the Osa Peninsula’s Golfo Dulce coastline, Costa Rica

**DOI:** 10.1371/journal.pone.0214390

**Published:** 2019-03-26

**Authors:** Beatriz Lopez Gutierrez, Angélica M. Almeyda Zambrano, Sandra L. Almeyda Zambrano, Carlos A. Quispe Gil, Stephanie Bohlman, Eduardo Avellan Arias, Guillermo Mulder, Clare Ols, Rodolfo Dirzo, Anneke M. DeLuycker, Karen Lewis, Eben N. Broadbent

**Affiliations:** 1 Spatial Ecology and Conservation Lab, School of Forest Resources and Conservation, University of Florida, Gainesville, Florida, United States of America; 2 Spatial Ecology and Conservation Lab, Department of Tourism, Recreation & Sport Management, University of Florida, Gainesville, Florida, United States of America; 3 Casa Rodden, Osa Peninsula, Costa Rica; 4 Lapa Rios Ecolodge, Osa Peninsula, Costa Rica; 5 Department of Biology, Stanford University, Stanford, California, United States of America; 6 Smithsonian-Mason School of Conservation, George Mason University, Washington D.C., United States of America; Montana State University, UNITED STATES

## Abstract

Habitat loss and fragmentation, together with related edge effects, are the primary cause of global biodiversity decline. Despite a large amount of research quantifying and demonstrating the degree of these effects, particularly in top predators and their prey, most fragmented patches are lost before their conservation value is recognized. This study evaluates terrestrial vertebrates in Playa Sandalo, in the Osa Peninsula of Costa Rica, which represents the last patch of “primary” forest in the most developed part of this region. Our study indicates that the diversity of ground species detected within Playa Sandalo rival other areas under active conservation like Lapa Rios Ecolodge. Historical fragmentation, together with the maintenance of forest cover in isolated conditions, are potentially responsible for the species composition observed within Playa Sandalo; facilitating the development of a prey-predator system including ocelots, medium-size mammals, and birds at the top of the trophic chain. The high diversity of both habitat and vertebrates, its prime location and cultural value, as well as its unique marine importance represent the ideal conditions for conservation. Conservation of Playa Sandalo, and other small tropical forest remnants, might represent the only management option for wildlife conservation within ever growing human-dominated landscapes.

## Introduction

Human activities have resulted in high deforestation and fragmentation of tropical forests. Since the 1990s, the total global tropical forest cover has decreased by 10% [1] and currently includes more than 50 million fragments [2]. Habitat loss, fragmentation, and patch isolation, compounded by edge effects [3], are the primary causes of global biodiversity decline [4,5]. The world´s wildlife populations are decreasing, while the rate of species extinctions is increasing, and loss is exacerbated by factors such as climate change [6]. It is estimated that more than 75% of Earth’s biodiversity has been already lost [7,8], and 3.5% more is expected to disappear by 2100, particularly in highly biodiverse tropical countries [8]. The rate at which both species and habitats are declining, together with the increasing number of forest fragments, highlight an impending need to evaluate biodiversity in forest patches, particularly to understand their value for conservation and, if appropriate, to apply required management actions before they are lost [9].

Although the consequences of anthropogenic impacts on biodiversity are well studied, the magnitude of change varies greatly among habitats and landscape context [10,11]. Similar forests can be influenced by distinct human activities driven, for example, by socio-economic factors unique to each area [5,12]. While some fragments may be subject to rapid or gradual degradation and ultimately habitat loss [13,14] other remnants can persist, both in time and space, with wildlife and humans coexisting in a human-dominated landscape [9,15]. For example, some forest patches might not be able to sustain a local population or an individual home range for certain species, such as large-size vertebrates; due to limited resources and connectivity or exposure to edge effects [3,12]. However, the same forest patches might maintain or increase populations of some species (e.g., small rodents) due to changes in predator-prey systems or reduced competition [16,17]. Some tropical forest patches serve as a refuge for migratory species [9,11,18]. The extent and complexity of ecological dynamics makes a standardized cross-site evaluation of biodiversity in forest patches challenging. Understanding fragments role in biodiversity conservation, as well as describing and comparing vertebrate species across distributed ranges, are considered priorities, particularly in biodiversity hotspots and for defaunation studies [19,20].

**Fig 1:**
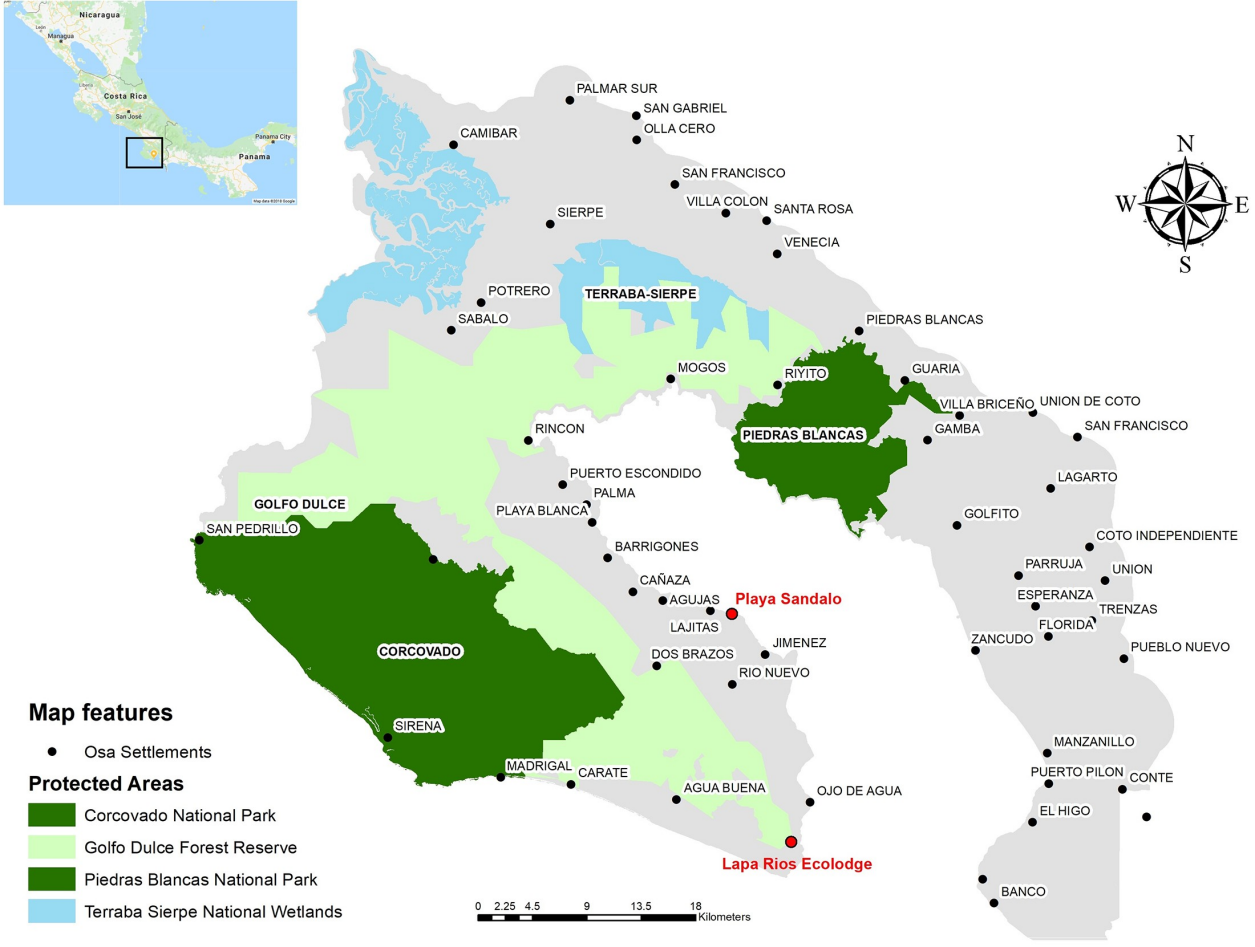
Map of the Osa Peninsula. The map included main settlements, Conservation Protected Areas (SINAC), Lapa Rios Ecolodge Nature Reserve and Playa Sandalo study locations (in red color). Also included location of the Peninsula within Central America. Matapalo, Lapa Rios Ecolodge area, not labelled in this map. Location Map Data: 2018 Google Maps.

The Osa Peninsula (Fig 1) is a priority area for conservation and considered to be one of the more biodiverse places on earth relative to its size (1093 km^2^), with 2.5% of the world’s living species [21,22]. It also has the most carbon-dense forests in the world [23]. Most of the Peninsula´s forest cover is safeguarded in a mosaic of natural protected areas and privately-owned areas. The region comprises three main public protected areas, Corcovado and Piedras Blancas National Parks, and the National Térraba Wetlands, several wildlife refuges, and private properties included in the Golfo Dulce Forest Reserve (GDFR) and areas adjacent to Golfo Dulce [24,25]. Ecotourism provides both environmental and socio-economic benefits in the Osa representing 60–80% of the economy [26,27]. Ecotourism initiatives, active reforestation, conservation, and regeneration resulting from land abandonment have resulted in a total of 80% forest cover over the entire peninsula, increasing at a rate of 5–6% yearly in some areas [24,28]. Although conservation is a strong trend in the Peninsula, hunting, gold mining, and industrial agriculture, such palm oil, persist in this region [29,30]. Outside of protected areas, information on the status of ground vertebrate species [31], hunting or human-predator conflict are scarce (www.inogo.info).

This study quantifies the ground terrestrial species (i.e. mammals and birds) within Playa Sandalo, a highly fragmented area containing the last patch of primary forest along the Golfo Dulce and compares the results with those obtained at Lapa Rios Ecolodge property, a Private Nature Reserve located within the GDFR which serves as a pseudo control (Fig 1). Both properties had historically similar forest conditions but are located in very different landscape matrixes with distinct land-use histories and land-tenure status (S1 Fig). The main objective of this study is to evaluate the ground terrestrial diversity of the Playa Sandalo area, highlighting its ecological and conservation potential. The descriptive comparison of indicator species carried out by this study is expected to highlight fragmentation of Playa Sandalo as a potential explanation for current composition of species; promoting and encouraging future assessments regarding general biodiversity patterns of this key fragmented area. Additionally, remote sensing analysis was used to evaluate and describe fragmentation and differences between environmental variables in both study areas (i.e. forest cover, size, location, and isolation with respect to forest edges, and the GDFR).

## Methods

### Study area

**Fig 2:**
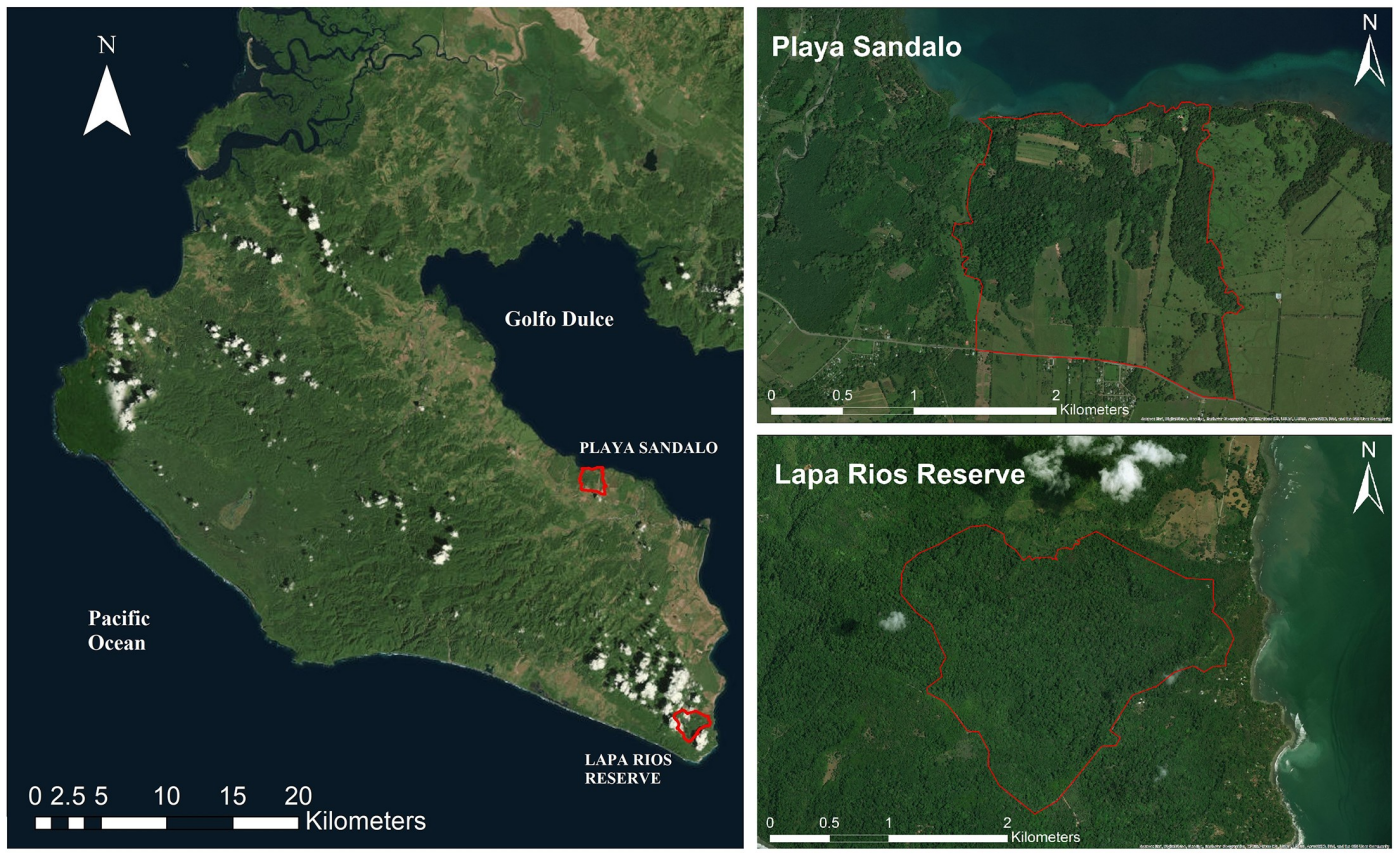
Study sites within the Osa Peninsula (Map Data: Esri, Digital Globe, GeoEye, Earthstar Geographics, CNES/Airbus, DS, USDA, USGS, AeroGRID, IGN, and GIS User Community). The Lapa Rios Reserve and Playa Sandalo property boundaries are highlighted with red marker lines. Depicted boundaries were created by this study using spatial features (i.e. roads and forest edges). Lapa Rios located within the GDFR (i.e. biological corridor), under strict conservation status since 1990s and serving as pseudo control group to compare with Playa Sandalo area. The later impacted by urban and agricultural development including multiple private land owners without legal protection status.

This research was conducted in the Osa Peninsula, Costa Rica. The Osa has a low population density (<7 people/km^2^). Puerto Jimenez is the main urban area with approximately 10,000 people living in or around the village [32]. Other population clusters comprise smaller settlements with a few hundred people or individuals living in remote farms. The climate in the Peninsula is hot and humid with an annual average temperature ranging from 17°C to 23°C, annual average precipitation of 4282mm and high levels of relative humidity varying between 88% and 98% [33]. Rainfall is more intense from August to early December (i.e. rainy season); followed by the dry season finishing in April. This research includes two areas within the Osa Peninsula, Lapa Rios Ecolodge Private Nature Reserve and Playa Sandalo (Fig 2). The study was carried out on private land with permission to conduct the study from the land owners.

#### Lapa Rios Ecolodge Natural Reserve (LRENR)

Lapa Rios is a privately-owned luxury ecolodge situated on the southwestern tip of the Osa Peninsula (N 8° 24' 0.9'', W 83° 17' 5.892'') (Figs 1 and 2). The property borders the Corcovado National Park and comprises approximately 4 km^2^ of lowland tropical rainforest included within the GDFR, part of the Mesoamerican Biological Corridor (www.sinac.go.cr). Lapa Rios has one of the largest private nature reserves in the country (RNVSO, Refugio Nacional de Vida Silvestre de Osa). Primary forests are dominant (80%) with some reforested and regenerated areas (20%), as well as a system of exploration trails running across the property and used for tourist activities. LRENR has been under rigorous protection and restoration since 1992 when the ecolodge was founded. Currently, it experiences high reforestation rates with nine times more increase in forest cover than the rest of the Peninsula [26] (S1 Fig). This area was isolated and had limited access until the 1950s when the existing dirt road was built and the Osa Forest Products (OFP) concession for forestry and mining was established in the area [34]. After that, significant deforestation and human pressures (i.e. hunting, cattle conflict) affected the area, until the 1990s when the Lapa Rios Reserve was established [27]. Currently, LRENR creates long-term ecological viability for key and endangered species such as jaguars (*Panthera onca*), peccaries (*Tayassu pecari* and *Pecari tajacu*), and tapirs (*Tapirus bairdii*), once limited to Corcovado National Park and now inhabiting, moving, or dispersing across the Reserve [31].

#### Playa Sandalo

Playa Sandalo is located northwest of the Peninsula (N 8° 34' 32.278'', W 83° 21' 32.802''). The Playa Sandalo area comprises approximately 3 km^2^ and it is owned, managed, and maintained by three private land owners. Most of the property is owned by two community members using the land for cattle ranching and farming. Independently, they have maintained forested areas, including a few areas of primary forest in the core of the property (i.e. around 20% of the forested area). The third portion is currently owned by an unknown United States land investor who contracts a local land owner to maintain it, principally though cattle ranching. Twenty years ago, the beachfront area, and much of the land in this area, was bought by a foreign investor who built a high-end vacation home, which after subsequent sales was purchased by the Rodden family from Georgia, United States, and which is now, called “Casa de Rodden” (currently on sale verified 09/10/2018). The Rodden property now encompasses 5 ha of lowland rainforest and mangroves, following sales of the other portions to local and international buyers in the 1990-2000s.

Playa Sandalo is the last patch of existing primary forest (Figs 1 and 2) along the Osa Peninsula’s Golfo Dulce shoreline extending from Rincon to the Lapa Rios Ecolodge. This area also represents the last marine zone with relative abundant live populations of coral reefs in the Golfo Dulce [35,36]. Its coastal and marine ecosystems are only a short distance from Puerto Jimenez, and have attracted locals and tourists to its beach, mangroves, and reef for generations. Playa Sandalo has become a popular tourist spot within the region, providing unique, accessible, and if tourists remain on the road–currently having no cost, natural ecosystems for bird watching and viewing fish on its reef. Accounts dating back to 1850 indicate the first economic developments for this area took place when Puerto Jimenez was established in 1914 [37]. Followed by the discovery of gold resources, urban development, roads, and multiple forestry and agricultural reforms, the Golfo Dulce area underwent rapid forest loss and degradation [24,25,34,38]. Playa Sandalo’s primary forest cover has however remained similar in shape, size, and isolation from 1969 to 2018 (S1 Fig), providing a remarkable and unique comparison of fragmented forest to the intact forest in less accessible areas of the peninsula and where the majority of research in this region has taken place.

### Study design

**Fig 3:**
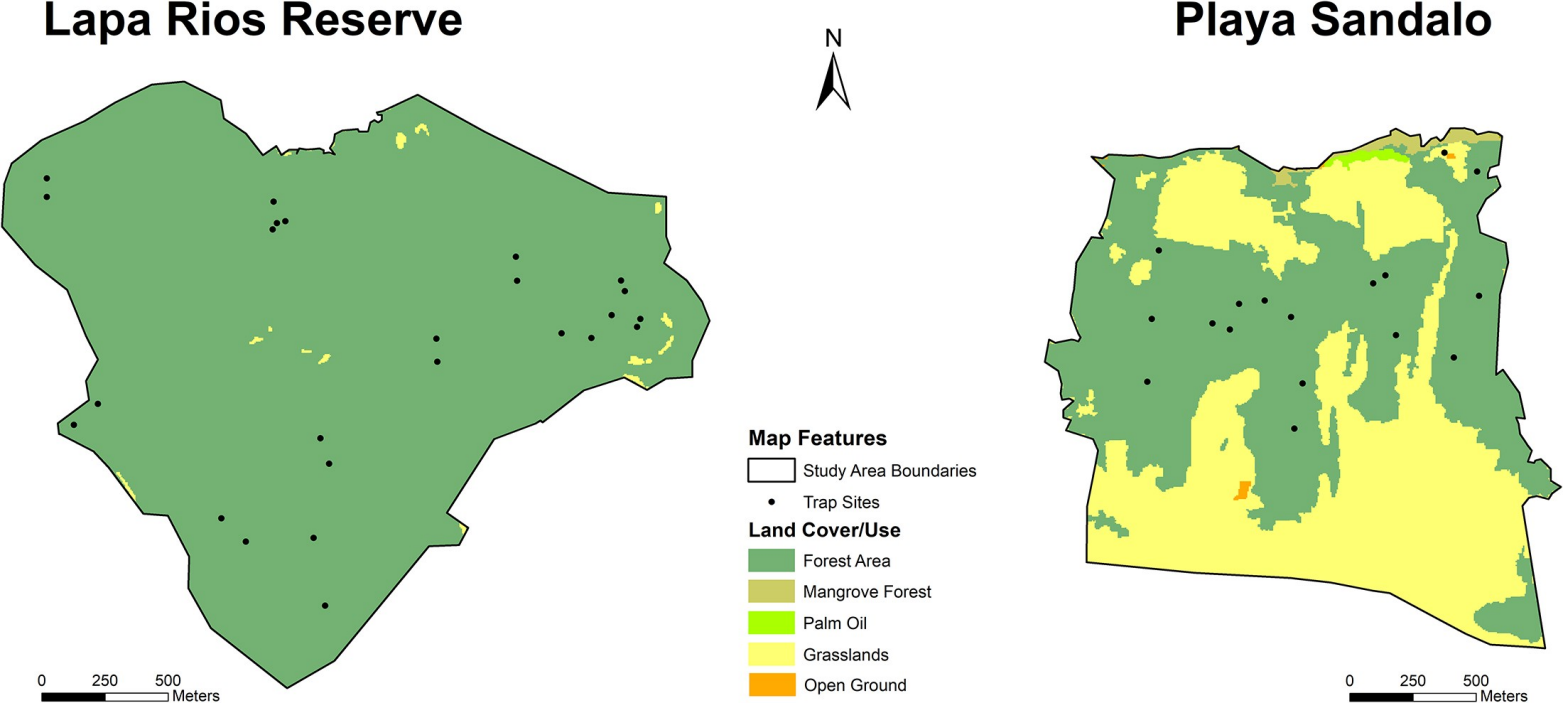
Trap sites and land cover/use within study area´s boundaries. Trap sites include 25 and 16 sites for Lapa Rios and Sandalo, respectively. High frequencies of dense forested areas, including mangrove forests in Playa Sandalo, and non-forested areas such as grasslands, palm oil, or open ground, were evaluated using land cover/use data provided by the Inogo Initiative (http://inogo.stanford.edu). Depicted boundaries were created by this study using spatial features (i.e. roads and forest edges).

Data was collected from April 22 to June 30, 2014, at LRENR and from May 20 to July 3, 2015, in Playa Sandalo, corresponding to the dry to rainy season transition (S1 File). Reconyx PC900 and Bushnell Trophy Cam HD Max cameras were used to measure terrestrial vertebrate species across primary and secondary forests in 25 sites in LRENR and 16 sites in Playa Sandalo (Fig 3). Camera placement at both areas followed two hierarchical criteria: 1) sites were determined at random using a map to represent the entire study area, and 2) specific locations with high animal activity features (i.e. trails) were favoured to maximize wildlife detection. At each location, the cameras were mounted roughly 0.3m-0.5m above the ground allowing the cameras to be triggered by motion from medium to large mammals as well as by small ground ones. Each camera recorded both during the day and night (24 hour-period) and included date and time at each trigger. GPS points were collected with a handheld navigational-grade Garmin receiver (Garmin Ltd, USA). Three cameras per study area were placed simultaneously and moved to different locations approximately every seven days maintaining a range of separation between sampling sites; 200 m for Playa Sandalo and 1000 m for Lapa Rios Reserve. These ranges were established according to forested areas available (Fig 2), as well as home ranges and camera trap protocols for evaluating tropical species expected within each study area (i.e. small-size in Playa Sandalo and medium-large size in Lapa Rios) [39]. During this time, cameras were checked for functionality and memory cards were replaced. In total, the collection periods at LRENR and Playa Sandalo were 176 and 110 trapping days respectively.

### Data analysis

Camera trap images were reviewed for photos containing an animal. An elapse of 30 minutes between consecutive photographs of the same species was determined to be an independent species event. In this way, each species is considered once regardless of the number of images taken within 30 minutes [40]. We recorded the date, time, and GPS coordinates for each event, while also identifying the species and noting the number of individuals at each event. Abundance was measured using the Species-specific Relative Abundance Index (RAI) [40,41]. The RAI was calculated by dividing the number of species events per site by the camera trap days per each site and then multiplying by 100, resulting in the standardized measure, number of events per 100 camera trapping days per site. We acknowledge the limitations of this procedure to estimate true measures of abundance or density [42]; however, we use RAIs as a standard procedure to compare both study areas [40,41,43].

Wilcoxon Rank-Sum Tests were used to compare Relative Abundance Index Values per site, specific species, and spatial variables between Lapa Rios and Playa Sandalo. Shannon Wiener Index and Jaccard´s and Sorensen´s Coefficients, using species events, were used to compare diversity and similarity between both study sites [15,44]. Shannon Wiener Index considers both the number of species present and their relative abundances to provide diversity and evenness indexes of the species present at each study site.

$$Shannon\;Wiener\;Index\;(H)=\sum_{i=1}^{N}pi\text{ln}\left(pi\right)$$

EvennessH=ln(N)/H

N = number of species

pi = proportion of N made up of species “i”

Alternatively, Jaccard´s and Sorensen’s coefficients use shared and site-specific species to produce a coefficient of similarity between both sites ranging from 0 to 1, from a dissimilar to similar.

$$Jaccard´s\;coefficient=\frac{a}{a+b+c}$$

$$Sorensen´s\;coefficient=\frac{2a}{2a+b+c}$$

a = shared species

b = specific species in Site 1

c = specific species in Site 2.

To evaluate relationships between the sampling effort and species richness at each study area, we generated rarefaction curves and species richness estimations using the package “vegan” [45] in R software version 3.5.2 [46]. Both procedures are widely used for biodiversity evaluations; assessing species-trapping effort dynamics while allowing standardization and comparison of datasets with different sampling efforts [47,48]. We excluded all domestic species (i.e. cows, pigs, and dogs), in addition to arboreal species (i.e. birds, primates, and squirrels) from these analyses [39]. In total six species were removed from Playa Sandalo and five from Lapa Rios.

Rarefaction analysis were conducted—with the R function “specaccum”—using species richness, frequency counts, and camera trapping sites per area to generate all possible accumulation curves drawn at random from re-ordering individuals per site within each sample [45]. The average refraction curve per study area was then calculated and plotted, along with 95% confidence intervals. Aditionally, we used the R function “poolaccum” to estimate extrapolated richness, or the number of undetected taxa (i.e. observed plus undetected taxa) from each area [45]. The first order Jackknife method was selected as the estimator for this analysis since it performs better than other widely used indices, for example Chao [49], for camera trap data [39,47]. This estimator calculated 100 randomizations (without replacement) from each given species pool; removing one or more observations from the sample set for each calculation, assuming there is no temporal variation in species capture probability and using the replicates to obtain expected asymptotic species richness for each study area [39].

### Spatial analysis

Boundary limits for Lapa Rios and Playa Sandalo were determined using visible features, including roads, paths, and land-use changes in recent satellite base map imagery available by ESRI (Figs 2 and 3). Forest percentage cover per site was calculated with Spatial Analyst Tools included in ArcMap (ArcGIS, ESRI, 2018, version 10.6) and a land-use map comprising land cover/use descriptions for the region available online and which represents the best and most updated currently available (http://inogo.stanford.edu). Five different buffers were selected around each camera trap—50, 100, 200, 300, and 500m—to evaluate immediate land cover/use changes surrounding the sites. The resulting land use within each buffer width was categorized into forested and non-forested areas. Forested areas include: any designation including dense forest cover (i.e. very high frequency for primary and high density for secondary forests), as well as mangrove forests within Playa Sandalo; non-forested areas include any agriculture (i.e. for livestock, woody, rice fields, or oil palm plantation.), open ground, water bodies, and infrastructure found within the buffers. Average forest cover percentages were calculated for each buffer, as well as per study area.

Sites, boundary limits, and buffer proportions were used to describe and highlight differences (i.e. vegetation, land cover/use, altitude, size, location, and isolation between both study areas. Boundaries were used to calculate study site size, whereas altitude per site was calculated using a Costa Rican Digital Elevation Model (DEM; SRTM 30m). Also, “distance to salient features” (i.e. roads, forest edges, and the GDFR) was measured using the ground distance calculator in Google Earth Pro version 6.0 to estimate each site´s isolation. Roads used to calculate “distance to salient feature” are described and represented by study area boundaries (Figs 2 and 3). Forest edge represented the transition from selected forested areas to any other land-cover type, for example, grassland, infrastructure, or palm oil, as well as any open ground area. When the land-use edge represented a gap within forested areas, only openings of more than 20 m^2^ were considered [50,51,52]. Additionally, a layer of the Conservation Areas of the Osa Peninsula produced by the National System of Conservation Areas (SINAC) was used to evaluate distance to the GDFR, part of the biological corridor connecting with mainland Costa Rica. A supplementary layer for the Golfo Dulce area was also created using the current forest cover and satellite images from Google Earth. Distances from the later and Playa Sandalo were also measured and used for comparison. The distance to the closest possible salient feature was selected for analysis. To examine and compare the effects of roads and forest edges on terrestrial vertebrates between and within study areas, relationships between wildlife and distances to salient features were evaluated using multivariate Spearman correlations.

## Results

**Fig 4:**
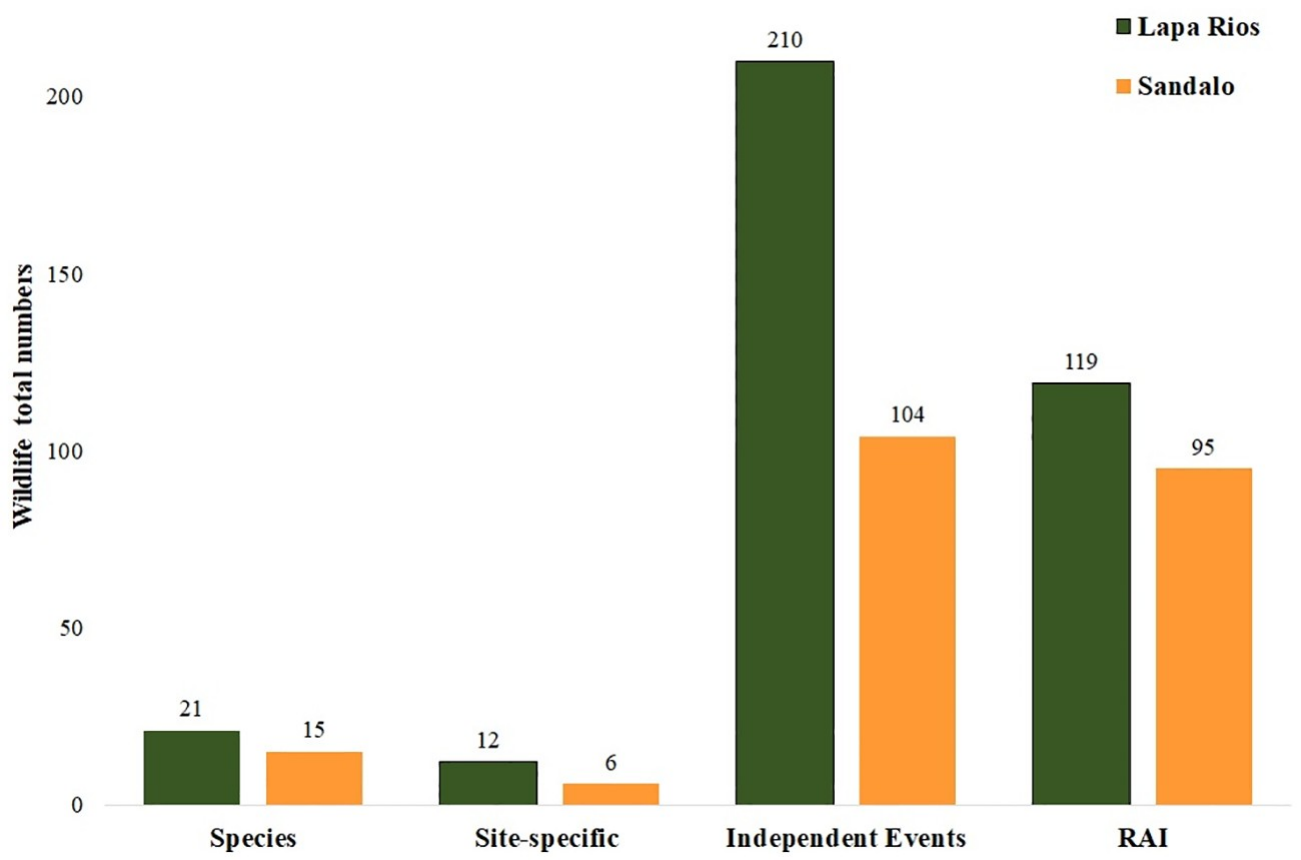
Total number of species, site-specific taxa, events, and RAI in Lapa Rios Ecolodge Nature Reserve and Playa Sandalo. Relative Abundance Index (RAI) = total events / trapping days *100. Domestic animals not included.

A total of 210 species events comprising 22 species were registered at LRENR: 21 wild species, and two independent events with domestic dogs (*Canis familiaris*). In contrast, there were 104 species events at Playa Sandalo including 18 species: 15 wild species and three domestic animals (dogs, cows, and pigs) (Table 1). LRENR had over 100 events more than Playa Sandalo; however, when events were standardized to trapping effort (i.e. RAI), the results present no significant differences for wildlife relative abundance between the two areas (Z = -0.97, p = 0.32) (Fig 4).

**Table 1:** Species independent events at Lapa Rios Ecolodge Nature Reserve and Playa Sandalo.

Group	Scientific Name	Common Name	Lapa Rios	Sandalo
MAMMALS				
Canidae	*Canis familiaris*	Domestic Dog	2	4
Bovidae	*Bos taurus*	Cow	-	2
Suidae	*Sus domesticus*	Domestic Pig	-	9
Felidae	*Panthera onca*	Jaguar	1	-
Felidae	*Leopardus pardalis*	Ocelot	2	1
Felidae	*Puma concolor*	Puma	4	-
Tayassuidae	*Pecari tajacu*	Collared Peccary	6	-
Tayassuidae	*Tayassu pecari*	White-lipped Peccary	7	-
Myrmecophaga	*Tamandua mexicana*	Northern Tamandua	1	2
Cebidae	*Ateles geoffroyi*	Spider Monkey	1	-
Cebidae	*Cebus capuchinus*	White-faced capuchin	-	1
Rodentia	*Dasyprocta punctata*	Agouti	92	-
Rodentia	*Cuniculus paca*	Paca	29	20
Dasypodidae	*Dasypus novemcinctus*	Nine-banded Armadillo	15	18
Dasypodidae	*Cabassous centralis*	Northern Naked-tailed Armadillo	1	-
Procyonidae	*Nasua narica*	White-nosed Coati	11	11
Procyonidae	*Procyon cancrivorus*	Crab-eating Raccoon	3	1
Procyonidae	*Procyon lotor*	Northern Raccoon	2	-
Didelphidae	*Didelphis marsupialis*	Common Opossum	1	7
Didelphidae	*Caluromys derbianus*	Central American Woolly Opossum	-	11
Mustelidae	*Eira barbara*	Tayra	1	-
Sciuridae	*Sciurus granatensis*	Red-tailed Squirrel	4	-
Sciuridae	*Sciurus variegatoides*	Variegated Squirrel	2	-
BIRDS				
Cracidae	*Crax rubra*	Great Curassow	22	-
Tinamidae	*Tinamus major*	Great Tinamou	4	3
Tinamidae	*Crypturellus soui*	Little Tinamou	-	15
Columbidae	*Leptotila cassinii*	Grey-chested Dove	1	2
Columbidae	*Leptotila verreauxi*	White-tipped Dove	-	1
Ardeidae	*Tigrisoma mexicanum*	Bare-throated Tiger Heron	-	1
Rallidae	*Aramides cajaneous*	Grey-necked Wood-rail	-	10

LRENR included 12 site specific taxa including large predators and their prey; for example, jaguars (*Panthera onca*), pumas (*Puma concolor*), or peccaries (*Tayassu pecari* and *Pecari tajacu*); while Playa Sandalo comprised more diversity of ground birds and smaller mammals, such as the Central American woolly opossum (*Caluromys derbianus*), representing half of the site-specific species found in this area (Table 1). There were no agoutis (*Dasyprocta punctata*) present at Playa Sandalo; however, there were more tamanduas (*Tamandua mexicana*) and common opossums (*Didelphis marsupialis*) detected at this location. Different species of primates were also detected at both locations: spider monkeys (*Ateles geoffroyi*) in Lapa Rios and white-face capuchins (*Cebus capuchinus*) in Playa Sandalo. Nine wildlife species were shared by both locations including nine-banded armadillos (*Dasypus novemcinctus*), and white-nosed coatis (*Nasua narica*). With similar event numbers pacas (*Cuniculus paca*) events were larger at LRENR; however, there were no significant differences between the relative abundance between both locations (Z = -0.39, p = 0.69). Despite the smaller size and being subject to more disturbance, Playa Sandalo showed a higher Shannon Diversity Index and Evenness than Lapa Rios (H = 2.26 vs 2.03; Evenness = 0.83 vs 0.67). Jaccard´s and Sorensen coefficients indicate distinct species composition for each studied area with scores of 0.33 and 0.50 respectively.

**Fig 5:**
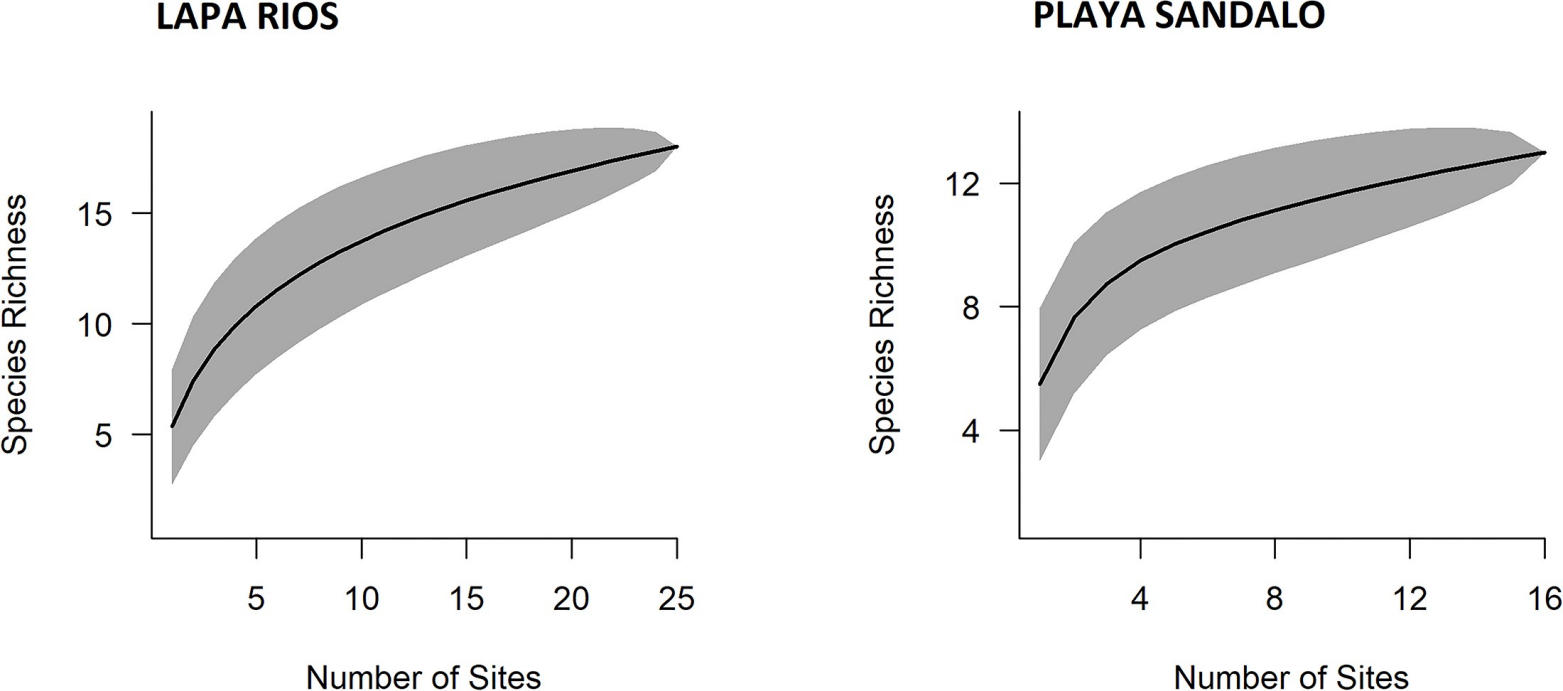
Rarefaction species-accumulation curves per camera trap site for Lapa Rios Ecolodge Nature Reserve and Playa Sandalo. Included average rarefaction curve and 95% confidence intervals based on species richness and frequency counts per site. The total data collected included: Lapa Rios (12 species and 90 individuals) and Playa Sandalo (17 species and 202 individuals).

The rarefaction curves, calculated from 12 species and 90 individuals for LRENR and 17 species and 202 individuals for Playa Sandalo, showed great variability per site as depicted by the confident intervals in Fig 5. Similar results for both areas were detected from the first camera trap site including 5.34 and 5.49 species for Lapa Rios and Playa Sandalo respectively. However, the curve from Playa Sandalo increased rapidly from the first 4 sites; accounting for 90% of the total richness observed within 7 trapping sites. In contrast, LRENR´s curve showed a steady increase, taking 17 sites to obtain 90% of the total richness sampled. None of the curves have reached a plateau, also determined by the estimation of species richness (Table 2). In total 71.04% of the species were detected for Playa Sandalo (i.e. 5 species undetected), whereas the percentage detection for LRENR was slightly lower (68.77%; 8 species undetected) (Table 2).

**Table 2:** Observed and estimated species richness in Playa Sandalo and Lapa Rios Ecolodge Nature Reserve. Included 95% confidence intervals and standard deviation calculated through the first order Jackknife method.

	Species Richness				
	Observed	Jack 1	Lower CI	Upper CI	SD
**Playa Sandalo**	12	16.89	15.73	17.68	0.95
**Lapa Rios**	17	24.72	19.79	25.66	1.49

The LRENR area comprised a total of 378 ha (3.77 km^2^), whereas Playa Sandalo included 298 ha (2.98 km^2^), as determined by the spatial analysis of the boundaries (Fig 3). Within those boundaries almost 100% of Lapa Rios Property was covered with primary and secondary forests. There were a few patches of shrub/woody grassland (< 20 m^2^) at the core of the Reserve, as well as other types of land use towards the edges—particularly at the north and east part—of the property. This is also highlighted by the buffer analysis of forest areas showing 98% cover even for 500m camera trap buffers (Table 3). In contrast, less than 50% of the area designated as Playa Sandalo was covered with forests including mangroves at the shores of Golfo Dulce. Main forested areas within Playa Sandalo were located at the core of the property, particularly further from the southern limit. Large areas of pasture for livestock were cultivated around Playa Sandalo forests, together with oil palm plantations adjacent to the shore (Fig 3). The main forest patches are connected by at least one > 20m forest strip; however, buffer analyses of Sandalo forested areas show that the forest cover is reduced drastically as the buffer area increased (Table 3).

**Table 3:** Average total percentage cover of forested and non-forested areas at different buffer widths in Playa Sandalo and Lapa Rios Ecolodge Nature Reserve. Forested areas comprise primary and secondary forests; additionally, mangrove forests are included for Playa Sandalo. Grasslands, palm oil, open ground, infrastructure and water correspond to non-forested areas.

Buffer	Playa Sandalo		Lapa Rios	
Width (m)	Forested	Non-Forested	Forested	Non-Forested
50	99.97	0.03	100	0
100	78.67	21.33	100	0
200	57.54	42.46	100	0
300	48.31	51.69	99.1	0.9
500	42.66	57.34	98.4	1.6

Average distances for measured features were compared for the study areas (Table 4). LRENR sites showed an average distance of 503m from the main road; whereas Playa Sandalo showed an average distance of 390m. There were no significant differences in road distances between both areas (Z = -0.44, p = 0.65). Alternatively, forest edge distances between LRENR and Playa Sandalo showed significant differences with averages of 413 and 118m, respectively (Z = -4.42, p < 0.001). Fifteen sites at LRENR were located within the GDFR boundary produced by SINAC. Thus, the distance to this feature was only 44m on average. The distance from GDFR to LRENR was reduced to zero when current forest cover boundaries were used. These represented significant differences with Playa Sandalo (Z = 5.46, p < 0.001) showing an average of 3679m from the GDFR when using established SINAC border designation. This distance was reduced by more than 500m by using current GDFR forest cover. Significant differences were proven using established and current borders between Playa Sandalo and GDFR (S = -68, p < 0.001). Alternatively, elevation between Lapa Rios and Playa Sandalo also showed significant differences with 16 and 213m, respectively; however, similar altitude values were included within each study area (Table 4).

**Table 4:** Lapa Rios Ecolodge Nature Reserve (LRNR) and Playa Sandalo (PS) elevation and distance (meters) to roads, forest edges, and the Golfo Dulce Forest Reserve (GDFR). Averages, maximum, and minimum values were included, as well as GDFR measurements: 1) GDFR (SINAC), borders of Conservation Areas by the National System of Conservation Areas, and 2) GDFR, representing current forest cover calculated by this study.

	Elevation		Road		Forest Edge		GDRF (SINAC)		GDRF	
	PS	LRNR	PS	LRNR	PS	LRNR	PS	LRNR	PS	LRNR
Aver	16	213	390	503	118	413	3679	44	3068	0
Max	24	302	753	1218	242	1046	4388	310	3919	0
Min	8	102	20	64	30	74	3123	0	2466	0

The relationships between wildlife relative abundance and selected variables for Lapa Rios and Playa Sandalo represent contrasting results. Wildlife relative abundance at Lapa Rios show negative correlations with roads (rs = -0.15, p > 0.1), forest edges (rs = -0.36, p < 0.1), and elevation (rs = -0.35, p < 0.1) (i.e. higher abundance with proximity or low elevation). Wildlife relative abundance at Playa Sandalo, in contrast, shows no significant relationships including more wildlife abundance further from edges (rs = 0.11, p > 0.1), roads (rs = 0.35, p > 0.1) and at points with “higher” elevation (rs = 0.28, p > 0.1).

## Discussion

The forests of Playa Sandalo are mostly located within 100m of an edge, where effects (i.e. altered microclimate, invasive species, pollution, erosion, etc.) are more severe [3,12]. Despite this fragmentation, the relative abundance of wildlife was similar to that of Lapa Rios Ecolodge Nature Reserve (Fig 4). In contrast, the species composition presented clear differences, with an absence of large-bodied animals, jaguars, pumas, peccaries, or tapirs, in Playa Sandalo.

Differences in species composition are in part due to large-bodied species being relatively abundant in Corcovado National Park and have dispersed and occupied areas within GDFR like Lapa Rios Ecolodge Natural Reserve [31]. Fragmented, isolated, and small forest patches, such as Playa Sandalo, cannot sustain populations of large-size vertebrates [12,53] without adequate connectivity. Limited resources and a constricting habitat decrease these species’ residency and increase predation [7,11], while the reduced connection with other fragments and the GDFR limits movement among fragments and recolonization [11,54]. In addition, the proximity and intensity of urban and agricultural development around the Playa Sandalo region increases edge effects, hunting pressures, and conflicts with cattle ranchers which might have potentially led to absence of animals such as felines, deer, tapir, or peccaries within Playa Sandalo [53,54,55]. The historical effects of hunting, together with their low fecundity [56], and diurnal activity patterns [31,57], might be responsible for the absence of sizable rodents and birds at Playa Sandalo. For example, agoutis or great curassows are considered a culinary delicacy across Latin America [55]. In addition, avian body size and responses to hunting can also exert stronger impacts on ground bird species than landscape fragmentation and structure [55,58]. Besides hunting, great curassows absence could be explained by their low occupancy in isolated forest patches in general, their forest dependency due to their granivore-frugivore diet, as well as their vulnerability to edge effects [39,55,58]. Despite these, the presence of these species was expected in Playa Sandalo where the size of forest cover and range of habitats—although not desirable due to size, degradation, and limited connection—should be enough to sustain small populations [53] (Figs 2 and 4). Agoutis, for example, are resilient creatures known to move across and adapt to human-altered landscapes [59]. Providing enough ground cover vegetation and in the absence of large predators, great curassows can also be found in plantation and partially open areas [60]. Additionally, both species include high detection probabilities for camera trap studies, particularly considering the small size of Playa Sandalo property [39,54]. Furthermore, large ground rodent and bird species are becoming common and abundant in other areas of the Peninsula [31] (B. Lopez Gutierrez, personal observation 2013–2018).

Alternatively, differences between large rodents and ground birds at Playa Sandalo could be related to body size influenced by the potential current carnivore trophic chain including ocelots, and potentially raptor birds as top predators [61]. These species prefer smaller prey, for example, tinamous, and woolly opossums, in great abundance in Playa Sandalo (Table 1). In the absence of top predators and other large species, paca´s population has remained relatively high; perhaps regulated by sporadic events of poaching or–according to locals—as a result of an illegal paca farm established in the area. The recolonization of top predators, such as jaguars, through increased connectivity, could have mixed consequences. Their estimated range includes 13 km^2^ for females and 50 km^2^ for males (J. C., Cruz Diaz, personal communication, 19 May 2018). Therefore, connecting Playa Sandalo with the GDFR—without previous protection and habitat restoration—could have significant consequences for the viability of the current prey-predator system established in this area. Increased predation and other impacts related to the introduction of high fecundity prey species such as peccaries could result in an abrupt decline of medium size vertebrates, thus the subsequent collapse of actual population dynamics [61]. We do see connectivity on the increase, with forest cover generally increasing in the Osa Peninsula [27] and the distance from Sandalo to GDFR appears to be reduced by 500m over the last decade (B., Lopez Gutierrez, personal observation).

The accumulation curves and richness estimators indicate that more effort per area, particularly spatial-temporal replication of sites, is required to provide accurate estimations of abundance, or to increase detection of rare species, habitat specialists, or animals with low detection rates [39,41,43] (Fig 5 and Table 2). For example, tapir (*Tapirus bairdii*) or deer (*Mazama americana*), were not detected during this evaluation, however they are present in Lapa Rios Ecolodge Natural Reserve [31]. Similarly, smaller species such as the greater grison (*Galictis vittata*) or tayras (*Eira barbara*) in Playa Sandalo, might have been present, but they were undetected by this study. In contrast, the presence of smaller taxa, or the great abundance of rodents, particularly in Playa Sandalo, could be related to a wide range of factors. For example, our sampling design, their high detection rates, specific home ranges, resource requirements, and/or foraging behaviour [39,41,43]. The underestimation or overestimation of species richness or abundance could result in bias when calculating relative abundance and diversity indexes [39,42].

Despite reservations and limitations resulting from the methodologies and the small scope of this study [39,42], species such as jaguars were detected in Lapa Rios, where larger forested areas and connection with GDFR should represent lower detection probability for this large and evasive species. In contrast, rodents such as agoutis with extremely high detectability rate, were absent in Playa Sandalo, while other rodents (i.e. pacas), tamanduas, or ocelots with lower detectability were represented [39,43]. Furthermore, estimates of species richness calculated by this study appear to be higher to the total number of taxa observed for the Peninsula (Table 2). Although, there are not published records for any of the areas assessed by this study, Lapa Rios has monitored their Natural Reserve for the last 4 years using 3 camera traps. So far, they have detected 21 species (G., Mulder, personal communication, 28 February 2019) suggesting that this rapid assessment was able to account for 80% of the totality of species observed in this area. Additionally, the only complete inventory of ground vertebrate species, currently under collection by the Osa Camera Trap Network and including 200 cameras across the region (http://osaconservation.org/projects/wildlife/osa-camera-trap-network/), has reported a total 25 species in the first three months of surveys (J. C., Cruz Diaz, personal communication, 3 March 2019). Although, the Osa Network species richness count correlates with our results for Lapa Rios, their inventory comprises the entire region detecting very rare species such as coyotes (*Canis latrans*); which have yet to be officially reported for the Osa Peninsula and whose presence is unlikely to occur in connected forested areas where Jaguars are present [62]. The presence of coyotes would be more likely to occur in fragmented landscapes such as Playa Sandalo; however, the presence of this or other larger species, as discussed above, is unlikely.

Remote sensing analyses highlight fragmentation as the most likely explanation for the detection of vertebrates in Playa Sandalo; which is supported by the comparison with LR. Playa Sandalo’s prime location, the social/cultural attachment and local willingness to conserve the area [26], particularly by the current owners, as well as its unique marine importance [35,36], represent the best conditions for conservation. The area has the potential to be used as a source of recolonization, both in terms of wildlife and habitat, facilitating and accelerating recovery of contiguous land and representing an economic alternative to restoration and the livelihood of current land owners [9,63,64].

Correlations with roads, edges, and elevation within study areas could be related detectability of species rather actual abundance [39,42]. In Playa Sandalo, our results are potentially related to the flat topography and fertile lowland soils; there is much agricultural development [24], particularly for livestock, causing habitat disturbance and increasing the intensity of edge effects. Degradation within the Osa Peninsula is more acute outside of the GDFR and close to urban areas surrounding the Golfo Dulce [4,25], where Playa Sandalo is located. Rarely studied and with no conservation efforts, these low and fertile forests are pressured, reduced, and degraded by urban and agricultural development [24]. Indeed, over the last few years, several large trees have been felled illegally in primary forest areas of Playa Sandalo (Dr. E.N, Broadbent, personal communication, 21, March 2018). This could have potential implications for both flora and fauna comprised in the area. Additionally, Casa Rodden is currently for sale highlighting how land tenure containing most forested areas could change at any point without guarantees. Without formal protection or left unmanaged, Playa Sandalo forests will remain both under constant degradation and with an unknown future.

## Conclusion and recommendations

Small forest patches with high biodiversity are key for the functioning of tropical ecosystems delivering services both at a local and a global scale. Besides their role as a reservoir of flora and fauna, they can greatly enhance water quality, are an important source for recolonization or carbon sink and can provide a set of cultural and economic services contributing to the development of community sustainable livelihoods and identity. Despite increased public and academic attention, forest fragments, particularly those embedded within agricultural and urban matrixes, have received little attention, as compared to public, large, legally protected, intact forest patches. Human-dominated landscape fragments are disappearing before their value for conservation is realized. This study found Playa Sandalo to represent a refuge for medium-size species such as pacas, tamanduas, primates, as well as for many other key smaller animals responsible for the biological and ecological functioning of tropical ecosystems [53]. These are also, potentially, influencing the vegetation structure, composition, and distribution [14,16]; ultimately affecting global ecosystem services [20].

Playa Sandalo´s diversity, regarding ground vertebrate species, rival other areas within the region under active conservation. This study indicates that fragmentation, lack of connectivity, and derived edge effects–among other factors–have potentially facilitated the development of a lower stable prey-predator system which could be intrinsically responsible for the current composition of species within this area. Playa Sandalo should be a priority area for conservation within the Osa Peninsula. It’s ground vertebrate diversity and potential arboreal composition (e.g., primates), its prime location and cultural value, as well as its unique marine importance, highlight its potential for conservation; a potential that without institutional and financial support, formal management and protection could be lost without the possibility of recovery.

This study provides baseline work for current and future data collection and spatial analysis occurring in this critical area of conservation. Other initiatives, such as the Osa Camera Trap Network comprising larger temporal and spatial scales, should consider occupancy modelling to evaluate occurrence, abundance, or other community features such as habitat use, while accounting for the probability of detection of specific species. This will allow more precise estimations of richness and abundance patterns within both areas. A generalized linear, or mixed, model is indicated to further evaluate relationships between environmental variables and wildlife patterns.

Information about reforested and regenerated areas and available spatial information in the region is scarce, preventing and limiting proportion estimations of primary, secondary and/or restored areas or the use of spatial autocorrelation analysis. The integration of traditional vegetation field surveys would complement available spatial information and further explore the results provided by this study. These vegetation variables could be then integrated with additional data from other key groups such as arboreal seed dispersers. For example, the authors are collaborating with a local NGO (Foundation Saimiri; www.fundacionsaimiri.org), working on research and conservation of monkey species in the region, to provide accurate information about diversity and behaviour of primate species within human-dominated landscape fragments such as Playa Sandalo. Extending the size of the buffers to further explore vegetation and/or land-cover use is also recommended. Including buffers of 1-10km, for instance, to represent animals with large ranges present at Lapa Rios, while extending the landscape component to assess fragmentation of Playa Sandalo at a larger scale.

Besides additional research opportunities described above, we also recommend 1) to protect, maintain and restore habitat so that current vertebrate populations can be maintained in Playa Sandalo and 2) to connect with the Golfo Dulce Forest Reserve allowing movement and reestablishment of larger species. A series of actions/initiatives promoting the development and completion of recommended stages are included in S1 Text. It is also recommended to revise the Golfo Dulce Forest Reserve legal boundary and conservation area to include forest cover increases produced in the last decade, to ensure forest connectivity in the future. Establishing a set of criteria which includes characteristics such as patch size, location, and isolation is also recommended to identify target locations for study and conservation. Existing forest patches in high biodiverse areas, particularly those including primary forests, could be evaluated using these criteria to be appropriately conserved before they are further degraded and fragmented.

## Supporting information

S1 Fig Land-cover change in the Osa Peninsula at different years, 1969, 1987, 1998, 2018 (Map Data: Google, US Geological Survey, Landsat / Copernicus, SIO, NOAA, U.S Navy, NGA, GEBCO, LDEO-Columbia, NSF). Lapa Rios Ecolodge went from deforestation to forest recovery after the Nature Preserve was stablished in 1992. In contrast, Playa Sandalo forest cover has remained in similar conditions since 1969. This image is for illustrate purposes only.
(TIF)

S1 Text Actions and initiatives for management of Playa Sandalo. (PDF)

S1 File Raw camera trap data collected in Lapa Rios Ecolodge Natural Reserve and Playa Sandalo. Included information from all photo events collected by this study (i.e. area, site, GSP location, date, time, species identification, and number of individuals).
(CSV)
