# Supplementary figures and images for: An island of wildlife in a human-dominated landscape: The last fragment of primary forest on the Osa Peninsula’s Golfo Dulce coastline, Costa Rica
Source: PLoS One. 2019 Mar 26;14(3):e0214390. doi: 10.1371/journal.pone.0214390 (PMC6435143; doi:10.1371/journal.pone.0214390)

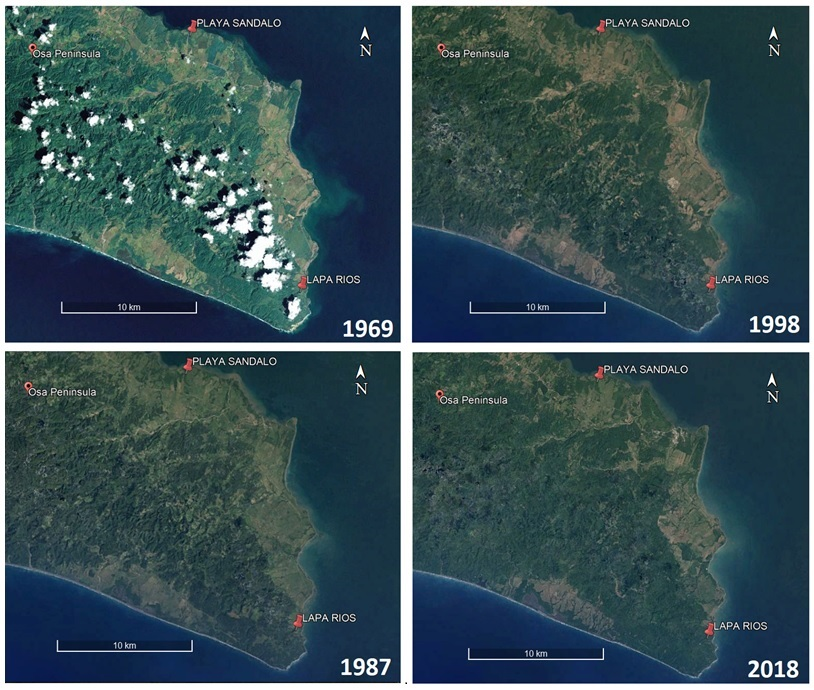

Supplement: S1 Fig — Lapa Rios Ecolodge went from deforestation to forest recovery after the Nature Preserve was stablished in 1992. In contrast, Playa Sandalo forest cover has remained in similar conditions since 1969. This image is for illustrate purposes only. (TIF) [file pone.0214390.s001.tif]
