# Supplementary material for: An island of wildlife in a human-dominated landscape: The last fragment of primary forest on the Osa Peninsula’s Golfo Dulce coastline, Costa Rica
Source: PLoS One. 2019 Mar 26;14(3):e0214390. doi: 10.1371/journal.pone.0214390 (PMC6435143; doi:10.1371/journal.pone.0214390)
Supplement: S1 Text — (PDF) [file pone.0214390.s002.pdf]

Two stages are recommended for management of Playa Sandalo: 1) to protect, maintain and restore habitat so that current vertebrate populations can be maintained, and 2) to connect with the Golfo Dulce Forest Reserve allowing movement and re-establishment of larger species.

1. Protect, maintain, and restore habitat and populations to maintain and increase existing vertebrate populations. The actions included here are aimed to promote these goals in the short term.

- Present the proposed study to representatives of SINAC, private institutions and stakeholders, as well as local landowners and communities, to showcase the importance of this area and promote the creation of Playa Sandalo Nature Sanctuary (http://www.playasandalo.org).
- Organising an open day and inviting local and regional representatives to visit and learn more about the area
- Searching and contacting national and international agencies, organisations, donors, as well as other potential sources of funding and support to buy Casa Rodden Property and assign legal conservation status.
- Provide information to local landowners on restoration and preventive conservation measures, as well as established legal initiatives such as Payment for Environmental Services, highlighting the benefits – economic and environmental - of adopting these strategies.
- Create trails, and other potential tourist attractions, and develop business's partnerships with local agencies and tour guides by which tourism is promoted and land owners incentivized.
- Utilize tourist services, visitors, and local organisations for reforestation and restoration efforts and/or to further our scientific knowledge of this area.

2. Connect with the Golfo Dulce Forest Reserve allowing movement and re-establishment of larger species. The following recommendations are key for successful completion of this stage.

- Habitat restoration and gradual reintroduction of absent large herbivory species to maintain diversity levels and ensure successful re-colonisation of top predators
- Restoration of exiting forested areas creating multiple wide passages and corridors to establish connectivity with the Golfo Dulce Forest Reserve.
- Identify and establish safe passages for wildlife to facilitate movement and safety of animals when crossing the main road, particularly at hot stops of mortality.
